# Prevalence of and factors associated with gestational diabetes mellitus and diabetes in pregnancy in Bangladesh: a facility-based cross-sectional study (2019–2020)

**DOI:** 10.1016/j.lansea.2026.100861

**Published:** 2026-09-10

**Authors:** Bishwajit Bhowmik, Tasnima Siddiquee, Shaila Parvin, Liya Quamrun Nesa, Shamima Akhtar, Shamim Khan, Faria Afsana, Tareen Ahmed, Faruque Pathan, Samsad Jahan Shelly, Kamrul Huda, Abul Kalam, Shukla Saha, Kaiser Alam Chowdhury, Mads Holst Jensen, AHM Enayet Hussain, Hajera Mahtab, Abul Kalam Azad Khan

**Affiliations:** a Centre for Global Health Research, Diabetic Association of Bangladesh, Shahbag, Dhaka, Bangladesh; b BIRDEM General Hospital & Ibrahim Medical College, Dhaka, Bangladesh; c National HealthCare Network, Diabetic Association of Bangladesh, Dhaka, Bangladesh

**Keywords:** Gestational diabetes mellitus, Diabetes in pregnancy, Hyperglycaemia in pregnancy, Prevalence, Screening, Bangladesh

## Abstract

**Background:**

Hyperglycaemia in pregnancy is associated with adverse maternal and offspring outcomes in South and Southeast Asia; however, nationally representative data from Bangladesh using contemporary diagnostic criteria remain limited. We aimed to estimate the nationwide prevalence of gestational diabetes mellitus (GDM) and diabetes in pregnancy (DIP), examine associated maternal factors, and assess the screening performance of the glucose challenge test (GCT).

**Methods:**

We conducted a nationwide facility-based cross-sectional survey (2019–2020) among pregnant women attending antenatal care across all administrative divisions of Bangladesh. A two-step screening strategy (50-g GCT followed by 75-g OGTT) was applied using WHO 2013 criteria. Multivariable logistic regression examined factors associated with GDM and DIP, and receiver operating characteristic analyses evaluated GCT performance and optimal cut-offs.

**Findings:**

Among 6631 women, the prevalence of GDM was 19.4% and DIP was 8.1%. Prevalence was higher in urban than rural areas for GDM (24.4% vs 12.6%) and DIP (11.0% vs 4.1%). Older age (≥25 years), urban residence, higher BMI, family history of diabetes, and physical inactivity (<30 min/day) were independently associated with both outcomes. Higher odds were observed with increasing number of maternal factors. The GCT showed good discrimination (AUC 0.83 for GDM; 0.89 for DIP).

**Interpretation:**

Hyperglycaemia in pregnancy affects a substantial proportion of women attending antenatal care in Bangladesh. The urban–rural gradient and clustering of modifiable risk factors, together with good GCT performance, support feasible integration of universal screening into routine antenatal care, acknowledging possible underestimation with the two-step approach.

**Funding:**

None.

**Research in context**

**Evidence before this study**

We searched PubMed, Scopus, and Google Scholar for studies published from database inception to May 1, 2026, using combinations of “gestational diabetes”, “diabetes in pregnancy”, “hyperglycaemia in pregnancy”, “prevalence”, “screening”, and “Bangladesh”, without language restrictions. Previous evidence was dominated by single-centre or urban studies using heterogeneous diagnostic criteria. A Bangladesh-specific meta-analysis of eight studies estimated a pooled GDM prevalence of 13%, but nationally comparable data covering urban and rural settings and evaluating both GDM and DIP remained scarce.

**Added value of this study**

This facility-based survey screened 6631 pregnant women attending 70 BADAS-affiliated antenatal centres across all 64 districts and eight administrative divisions using a standardised two-step pathway. It provides geographically broad estimates of GDM and DIP, quantifies urban–rural and divisional variation, identifies associated maternal factors, and evaluates the discriminatory performance of the 50-g GCT in a resource-constrained setting.

**Implications of all the available evidence**

Together with previous evidence, our findings indicate a substantial burden of hyperglycaemia in pregnancy in Bangladesh and support consistent implementation of standardised screening within routine antenatal care. The results also support strengthening referral, diagnostic, and follow-up capacity while addressing modifiable maternal factors and evaluating implementation in population-based and primary-care settings.

## Introduction

Gestational diabetes mellitus (GDM), defined as hyperglycaemia first detected during pregnancy, is associated with adverse maternal and perinatal outcomes, including pre-eclampsia, macrosomia, neonatal hypoglycaemia, and a substantially increased lifetime risk of type 2 diabetes (T2D) for both mother and offspring.^1^^,^^2^ Recent global estimates indicate that hyperglycaemia in pregnancy (HIP) is common and increasing, with GDM accounting for the majority of cases worldwide.^3^ In South and Southeast Asia, including Bangladesh, rapid urbanisation, dietary transition, rising adiposity, and declining physical activity are thought to be major contributors; however, reported prevalence varies widely across studies.^3^, ^4^, ^5^, ^6^

A key source of this variability is methodological heterogeneity. A Bangladesh-specific systematic review and meta-analysis including eight studies (n≈6948) reported a pooled GDM prevalence of 13% (95% CI 7–21) and identified higher prevalence among older and obese mothers.^4^ However, differences in diagnostic strategies—particularly the use of the International Association of the Diabetes and Pregnancy Study Groups (IADPSG)/WHO-2013 one-step 75-g oral glucose tolerance test (OGTT) criteria compared with older WHO-1999 or Carpenter–Coustan thresholds—substantially limit comparability, as studies applying IADPSG/WHO-2013 criteria consistently identify more cases.^5^^,^^6^ Moreover, much of the existing evidence in Bangladesh is derived from hospital-based, predominantly urban populations, restricting generalisability and limiting understanding of urban–rural differences in GDM burden. In Bangladesh, approximately 43% of the population resides in urban areas, reflecting rapid demographic transition that may influence geographic variation in GDM burden.^7^

In Bangladesh, national guidance adapted from WHO recommendations supports testing for hyperglycaemia in pregnancy at 24–28 weeks’ gestation, with earlier testing for women at high risk. In practice, screening is delivered through antenatal services in government, non-governmental, and private facilities, but access to standardised testing and implementation vary by facility level, laboratory capacity, and geography. Consequently, screening is not yet implemented uniformly across the national antenatal-care platform.^8^^,^^9^

Emerging data suggest that such geographic gradients may reflect underlying distributions of established factors, including maternal age ≥25 years, higher BMI, family history of T2D, and hypertension, all of which have been associated with dysglycaemia in pregnancy in South Asian populations.^4^^,^^10^, ^11^, ^12^ However, nationally comparable data using standardised diagnostic pathways and covering both urban and rural populations remain limited in Bangladesh.

Accordingly, we conducted a facility-based cross-sectional survey across all 64 districts of Bangladesh to estimate the prevalence of GDM and diabetes in pregnancy (DIP) among women attending antenatal care, and to examine associated sociodemographic, lifestyle, and clinical factors. We further evaluated the discriminatory performance of a two-step screening strategy comprising a 50-g glucose challenge test (GCT) followed by a 75-g OGTT interpreted according to IADPSG/WHO-2013 criteria.

We examined whether prevalence differed by maternal age, BMI category, family history of T2D, hypertension status, and urban versus rural residence, using two-sided hypothesis testing throughout.

## Methods

We conducted a facility-based cross-sectional survey across all 64 districts of Bangladesh from November 2019 to October 2020, using the WHO STEPS instrument (structured questionnaire, physical measurements, and biochemistry protocols) to standardise data collection, procedures, and quality control.^13^ This study did not implement the full population-based STEPwise surveillance approach but used selected components to ensure methodological consistency across sites.

Data collection was coordinated through 70 Diabetic Association of Bangladesh (BADAS)-affiliated centres, purposively selected to ensure geographic representation across all administrative divisions, including both urban and rural populations, based on predefined criteria such as geographic coverage, patient volume, and capacity to perform standardised testing procedures.

BADAS centres serve as general healthcare facilities open to all community members rather than specialised centres for high-risk populations. As the largest non-governmental primary healthcare provider in Bangladesh, BADAS manages a substantial proportion of patients with diabetes while also delivering routine antenatal care, general outpatient services, and non-communicable disease screening. Services are provided on a walk-in basis without restriction to individuals with known diabetes or elevated risk, thereby facilitating recruitment from a broad population base.^14^

Participant recruitment was conducted consecutively among eligible pregnant women attending routine antenatal care at participating centres during the study period, without selective enrolment.

Consecutive pregnant women attending routine antenatal care at participating centres were screened. Eligibility criteria were age 18–45 years, gestational age 24–28 weeks and written informed consent. Women with known pre-pregnancy diabetes or acute illness requiring hospitalisation were excluded.

Screening was conducted at 24–28 weeks’ gestation in accordance with IADPSG/WHO recommendations; early gestational diabetes diagnosed before 20 weeks was not assessed. Participant recruitment, exclusions, and analytical flow are reported in accordance with STROBE item 13.^15^

All participants were pregnant women attending antenatal care services; therefore, sex was uniform across the study population and was not included as an analytical variable. Data on gender identity were not collected. Ethnicity was also not collected because the study was designed as a nationwide survey of pregnant women attending antenatal care facilities in Bangladesh, where the population is predominantly ethnically Bengali.

A standardised two-step screening pathway was implemented at 24–28 weeks’ gestation.

This approach was adopted for pragmatic reasons in a resource-constrained setting, where universal OGTT screening is logistically challenging due to cost, fasting requirements, and laboratory capacity.

Step 1 (screening): a 1-h 50-g GCT was performed irrespective of fasting status. Capillary glucose was measured using calibrated digital glucometers, and a value ≥7.8 mmol/L was considered a positive screen, prompting referral for confirmatory testing. Women with a negative GCT were not referred for OGTT.
Step 2 (diagnosis): a 75-g OGTT was conducted after an 8–14-h overnight fast, with venous plasma glucose measured at fasting and 2 h after 75 g glucose load. GDM was defined using IADPSG/WHO-2013 criteria as fasting plasma glucose (FPG) ≥5.1 mmol/L and <7.0 mmol/L and/or 2-h plasma glucose (2 hPG) ≥8.5 mmol/L and <11.1 mmol/L. DIP was defined as FPG ≥7.0 mmol/L and/or 2 hPG ≥11.1 mmol/L, consistent with WHO/IADPSG recommendations.^16^^,^^17^

Venous plasma glucose was measured using the glucose oxidase method in participating laboratories. Internal quality control procedures included duplicate assays on 10% of samples, with intra- and inter-assay coefficients of variation maintained below 2% throughout the study period. The use of capillary blood glucose for initial screening has been validated in the Bangladeshi population, demonstrating strong concordance with venous plasma glucose measurements in prior studies.^18^ Glucometers were plasma-calibrated and calibrated according to manufacturer specifications and site standard operating procedures.

Trained interviewers administered a structured questionnaire capturing sociodemographic characteristics (age, education, household expenditure, residential setting), behavioural factors (tobacco exposure, diet, physical activity), and obstetric history (parity, previous pregnancy complications).

Anthropometric measurements (weight, height) and blood pressure were obtained at 24–28 weeks’ gestation using standardised protocols and calibrated equipment. Body mass index (BMI) was calculated at mid-gestation; pre-pregnancy BMI was not available. BMI was calculated as weight (kg)/height (m^2^), and general obesity was defined as BMI ≥25 kg/m^2^.^19^ Hypertension (HTN) was defined as systolic blood pressure ≥140 mmHg, diastolic blood pressure ≥90 mmHg, or current antihypertensive use.^20^

Socioeconomic status was classified using monthly household expenditure as low (<10,000 Bangladeshi Taka [BDT]), medium (10,000–20,000 BDT), or high (>20,000 BDT). Residential status was coded as urban or rural according to national administrative definitions.^21^ Education was grouped into 0–5 years, 6–10 years, and >10 years of schooling.

Physical activity was assessed from self-reported daily walking duration. The Global Physical Activity Questionnaire (GPAQ) was not used, and MET-minutes were not calculated. Physical activity was categorised as inactive (<30 min/day) versus active (≥30 min/day), consistent with prior regional studies.^22^^,^^23^

Dietary intake was summarised as self-reported frequency of fruit and vegetable consumption. Inadequate intake was defined as consumption on fewer than 7 days per week.

Preconception care was defined as at least one health consultation or intervention before pregnancy aimed at optimising maternal health, as self-reported by participants.

The sample size was determined to estimate the prevalence of gestational diabetes mellitus (GDM) with adequate precision. Based on prior evidence from Bangladesh, including a systematic review and meta-analysis, the expected prevalence was assumed to be 9.7%.^10^ A single population proportion formula was applied with a 95% confidence level (Z = 1.96) and an absolute precision of 1%:

$$n=\frac{Z^{2}\times p\times \left(1-p\right)}{d^{2}}$$

where p = 0.097 and d = 0.01. This yielded a minimum required sample size of 3365 participants. To account for the multicentre design and potential clustering across study sites, a design effect of 1.2 was applied, increasing the sample size to 4038. Assuming a 10% non-response or exclusion rate, the final minimum required sample size was estimated at approximately 4442 participants.

All eligible pregnant women attending participating centres during the study period were recruited consecutively without imposing an upper sample size limit, in line with the facility-based design of the study. This approach was adopted to maximise statistical precision and ensure adequate representation across geographic regions.

A total of 6631 participants were ultimately included in the analysis, substantially exceeding the minimum required sample size. This increased sample size enhanced the precision of prevalence estimates and statistical power for subgroup and multivariable analyses.

Although a design effect was incorporated at the planning stage, clustering at the centre or district level was not explicitly modelled in the final analysis, which may have resulted in underestimation of standard errors.

Selection bias was minimised by recruiting consecutive attendees across geographically diverse centres. However, as this was a facility-based study, participants may not fully represent the general pregnant population. Measurement bias was reduced through standardised training, site initiation visits, and regular equipment calibration. Laboratory bias was minimised through duplicate testing and continuous monitoring of assay performance. Data were double entered, with range and logic checks applied prior to analysis.

### Statistical analysis

Analyses were performed using Stata version 14.2 (StataCorp, College Station, TX, USA), IBM SPSS Statistics version 21.0 (IBM Corp., Armonk, NY, USA), and Python version 3.9 (Python Software Foundation, Wilmington, DE, USA). Prevalence estimates were calculated for GDM and DIP overall and by prespecified subgroups. Continuous variables are presented as mean (standard deviation [SD]) and categorical variables as percentage (number). Continuous variables were assessed for approximate normality before parametric analyses.

Group comparisons used χ^2^ tests for categorical variables and independent t-tests or one-way analysis of variance (ANOVA) for continuous variables, as appropriate.

Univariable and multivariable logistic regression models were used to estimate crude and adjusted odds ratios (ORs) with 95% confidence intervals (CIs) for factors associated with GDM and DIP. For categorical variables with more than two categories, overall statistical significance was assessed using Wald tests, whereas odds ratios were estimated for individual categories relative to the reference category. These analyses were prespecified and exploratory in nature given the cross-sectional design; therefore, associations are interpreted as non-causal.

As a sensitivity analysis, multivariable models were re-estimated after excluding hypertension because of its potential position on the causal pathway between adiposity and hyperglycaemia in pregnancy. Results were materially unchanged and are therefore not presented.

Covariates were selected a priori based on established literature on GDM risk factors in South Asian populations, including age, BMI, family history of type 2 diabetes, hypertension, parity, residential setting, education, and physical activity. Hypertension was included in the primary multivariable models but evaluated separately in sensitivity analyses. Parity was not retained in the final multivariable models because it was not associated with either outcome in univariable analyses. Dietary variables were retained in the multivariable models because of their epidemiological relevance despite limited statistical significance.

Multicollinearity among covariates was assessed using variance inflation factors (VIFs), with all VIF values below 5, indicating no evidence of problematic multicollinearity. Because missing data were minimal (<5% for all variables), complete-case analysis was performed without imputation. All statistical tests were two-sided, and p values < 0.05 were considered statistically significant.

Diagnostic performance of the GCT was evaluated using receiver operating characteristic (ROC) curve analysis. Areas under the ROC curve (AUCs) with 95% CIs were calculated separately for GDM and DIP. Optimal GCT cut-off values were identified using the Youden index, and corresponding sensitivity and specificity estimates were derived. Additionally, positive predictive value (PPV) and negative predictive value (NPV) were calculated for these optimal cut-offs.

The OGTT (FPG and 2 hPG) served as the reference standard for evaluating GCT performance. ROC analysis for OGTT itself was not performed, as this would require an independent clinical outcome or external gold standard beyond the scope of this cross-sectional study.

Cumulative maternal risk was assessed using an additive approach by sequentially combining independent factors identified in multivariable models. Adjusted odds ratios were used to illustrate increasing likelihood (rather than causal risk) of GDM and DIP with accumulation of these factors. These analyses were visualised graphically (Fig. 3B and C).

### Ethics statement

Ethics approval was obtained from the BADAS Ethical Review Committee (BADAS-ERC/EC/17/0133). Written informed consent was obtained from all participants (witnessed thumbprint for participants unable to sign). Data were anonymised before analysis. The study was conducted in accordance with the Declaration of Helsinki and its subsequent amendments.

### Role of funding source

This study received no funding.

## Results

A total of 6631 pregnant women were included in the final analysis between November 2019 and October 2020. Overall, 4812 (72.5%) women had normoglycaemia, 1285 (19.4%) had GDM, and 534 (8.1%) had DIP. Missing data for covariates were <5%; complete-case analyses were applied. Denominators for each variable are provided in the tables to ensure transparency regarding item-level missingness.

Baseline characteristics by glycaemic category are presented in Table 1. Compared with normoglycaemic women, those with GDM or DIP were older, with a higher proportion aged ≥25 years (p < 0.001), and were more likely to reside in urban areas (p < 0.001). Higher educational attainment and greater monthly household expenditure were also more common among women with GDM and DIP (both p < 0.001).

**Table 1:** Baseline characteristics of study participants by glycaemic status.

Variable	Total	Normal	GDM	DM in pregnancy	P value
% (Number)	6631	72.5 (4812)	19.4 (1285)	8.1 (534)	
Age (years)^a^	25.4 (5.3)	24.6 (5.1)	27.2 (5.2)	28.2 (5.5)	<0.001
Age group^b^					
<25 years	45.3 (3001)	51.8 (2492)	29.8 (383)	23.6 (126)	<0.001
≥25 years	54.7 (3630)	48.2 (2320)	70.2 (902)	76.4 (408)	
Education (years)^b^					
0–5	26.2 (1656)	28.4 (1307)	20.7 (253)	19.5 (96)	<0.001
6–10	43.3 (2736)	43.8 (2014)	42.0 (514)	42.2 (208)	
>10	30.5 (1926)	27.8 (1280)	37.3 (457)	38.3 (189)	
Occupation^b^					
Housemaker	80.3 (5328)	81.4 (3917)	77.4 (995)	77.9 (416)	0.002
Others	19.7 (1303)	18.6 (895)	22.6 (290)	22.1 (118)	
Monthly expense (BDT)^b^					
Low income (<10,000)	40.7 (2592)	44.2 (2048)	32.5 (400)	28.4 (144)	<0.001
Middle income (10,000–20000)	44.5 (2831)	43.7 (2021)	45.4 (558)	49.7 (252)	
High income (>20,000)	14.8 (944)	12.1 (561)	22.1 (272)	21.9 (111)	
Location^b^					
Rural	42.3 (2807)	48.6 (2339)	27.5 (353)	21.5 (115)	<0.001
Urban	57.7 (3824)	51.4 (2473)	72.5 (932)	78.5 (419)	
Smoking habit^b^	2.2 (134)	1.8 (81)	3.2 (38)	3.1 (15)	0.005
Vegetables/7 days^b^	28.9 (1915)	27.0 (1297)	32.5 (417)	37.6 (201)	<0.001
Fruits/7 days^b^	13.6 (899)	11.8 (570)	19.5 (250)	14.8 (79)	<0.001
Physical activity^b^					
≥30 min	52.0 (3038)	56.5 (2398)	40.9 (458)	38.4 (182)	<0.001
<30 min	48.0 (2799)	43.5 (1844)	59.1 (663)	61.6 (292)	
Number of parity (≥1)^b^	37.5 (1563)	37.0 (1079)	37.6 (327)	41.0 (157)	0.31
Preconception care^b^	34.0 (2114)	32.8 (1477)	36.2 (435)	39.8 (202)	0.001
Previous Pg complications^b^	24.6 (987)	22.5 (630)	29.0 (245)	30.1 (112)	<0.001
Use of OCP^b^	39.2 (2374)	39.7 (1750)	35.9 (418)	42.0 (206)	0.025
Family history of diabetes^b^	33.4 (2171)	28.2 (1331)	45.6 (573)	50.6 (267)	<0.001
Weight (kg)^a^	58.4 (10.4)	56.8 (9.8)	61.9 (11.0)	63.9 (10.1)	<0.001
BMI (kg/m^2^)^a^	25.2 (4.3)	24.6 (4.2)	26.4 (4.6)	27.2 (4.0)	<0.001
BMI (kg/m^2^) categories^b^					
Underweight (<18.5)	4.1 (253)	5.0 (224)	2.0 (24)	1.0 (5)	<0.001
Normal weight (18.5–22.9)	28.4 (1768)	32.4 (1456)	21.2 (259)	10.7 (53)	
Overweight (23–24.9)	20.5 (1275)	21.4 (961)	17.8 (217)	19.5 (97)	
Obese (≥25)	47.0 (2919)	41.3 (1855)	59.1 (722)	68.8 (342)	
SBP (mmHg)^a^	113.5 (13.2)	112.3 (13.2)	115.9 (12.7)	117.8 (12.9)	<0.001
DBP (mmHg)^a^	74.0 (8.6)	73.3 (8.7)	75.5 (7.9)	76.5 (8.6)	<0.001
HTN^b^	12.7 (845)	12.3 (590)	12.8 (163)	17.2 (92)	0.005
1 h CBG (mmol/l)^a^	7.8 (2.1)	6.9 (1.8)	9.5 (2.1)	12.0 (3.1)	<0.001
FPG (mmol/l)^a^	5.8 (1.6)	4.4 (0.5)	5.8 (0.6)	7.9 (2.1)	<0.001
2 hPG (mmol/l)^a^	9.2 (2.8)	6.7 (1.1)	8.9 (1.3)	13.3 (2.9)	<0.001

BDT, Bangladeshi taka (1 USD = 120 BDT); BMI, body mass index; CBG, capillary blood glucose; DBP, diastolic blood pressure; DM, diabetes mellitus; F/H, family history; FPG, fasting plasma glucose; GDM, gestational diabetes mellitus; 2 hPG, 2 h after plasma glucose; HTN, hypertension; OCP, oral contraceptive pill; SBP, systolic blood pressure.

a Data are presented as mean (SD) as needed.

b Data are presented as percentage (number) as needed.

Cardiometabolic profiles differed across glycaemic categories. Women with GDM or DIP had higher mean BMI and body weight (both p < 0.001), as well as higher systolic and diastolic blood pressure (p < 0.001). Glycaemic measures, including 1-h capillary glucose, FPG, and 2-h OGTT values, showed a progressive increase across categories from normoglycaemia to GDM and DIP (all p < 0.001). BMI category distributions demonstrated a graded increase in overweight and obesity across glycaemic groups (p-trend <0.001).

Daily physical activity <30 min was more common among women with GDM and DIP (p < 0.001). Fruit intake (p < 0.001) and vegetable intake (p < 0.001) differed across glycaemic categories. Family history of diabetes (p < 0.001), preconception care attendance (p = 0.001), previous pregnancy complications (p < 0.001), and oral contraceptive pill use (p = 0.025) also differed significantly across groups.

Parity did not differ significantly across glycaemic categories (p = 0.314), whereas hypertension was more common among women with DIP than among normoglycaemic women (p = 0.005) (Table 1).

Supplementary Table S1 presents prevalence estimates of GDM and DIP across participant characteristics; p-values compare women with GDM and DIP separately against normoglycaemic participants. Both GDM and DIP were more prevalent among women aged ≥25 years, those with higher educational attainment, greater household expenditure, urban residence, lower physical activity, obesity, and a family history of diabetes (all p < 0.001).

GDM prevalence was higher among women reporting lower fruit intake (p < 0.001), lower vegetable intake (p = 0.002), and previous pregnancy complications (p = 0.001). DIP prevalence was higher among women with hypertension (p = 0.001), previous pregnancy complications (p = 0.009), and no preconception care (p = 0.004). Underweight women had the lowest prevalence, with a dose–response pattern observed across normal weight, overweight, and obesity categories (p-trend <0.001).

Results of the univariable and multivariable logistic regression analyses are presented in Table 2. After adjustment, older maternal age, urban residence, obesity, family history of diabetes, and physical activity of less than 30 min per day remained independently associated with both GDM and DIP. The magnitude of association with obesity and urban residence was greater for DIP than for GDM. Other sociodemographic, dietary, clinical, and reproductive factors were not independently associated with either outcome.

**Table 2:** Crude and adjusted odds ratios for factors associated with gestational diabetes mellitus (GDM) and diabetes in pregnancy (DIP).

Risk indicator	GDM			P value	DIP			P value
	Crude OR (95% CI)	P value	Adjusted OR (95% CI)		Crude OR (95% CI)	P value	Adjusted OR (95% CI)	
Age group								
<25 years	Reference		Reference		Reference		Reference	
≥25 years	2.5 (2.2, 2.9)	<0.0001	1.8 (1.5, 2.4)	<0.0001	3.5 (2.8, 4.3)	<0.0001	1.8 (1.3, 2.5)	<0.0001
Education (years)		<0.0001		0.29		<0.0001		0.82
0–5	Reference		Reference		Reference		Reference	
6–10	1.3 (1.1, 1.6)		1.1 (0.8, 1.4)		1.4 (1.1, 1.8)		1.3 (0.9, 1.9)	
>10	1.8 (1.6, 2.2)		1.2 (0.9, 1.6)		2.0 (1.6, 2.6)		1.2 (0.8, 1.7)	
Occupation								
Housemaker	1.2 (1.1, 1.3)	0.004	1.0 (0.9, 1.2)	0.96	1.2 (1.1, 1.4)	0.013	0.9 (0.8, 1.2)	0.94
Others	Reference		Reference		Reference		Reference	
Monthly expense (BDT)		<0.0001		0.029		<0.0001		0.18
Low income (<10,000)	Reference		Reference		Reference		Reference	
Middle income (10,000–20000)	1.4 (1.2, 1.6)		1.3 (1.1, 1.7)		1.8 (1.4, 2.2)		1.2 (0.9, 1.7)	
High income (>20,000)	2.5 (2.1, 2.9)		1.4 (1.1, 2.0)		2.8 (2.2, 3.7)		1.5 (0.9, 2.2)	
Location								
Rural	Reference		Reference		Reference		Reference	
Urban	2.5 (2.2, 2.9)	<0.0001	2.2 (1.7, 2.7)	<0.0001	3.4 (2.8, 4.3)	<0.0001	2.5 (1.8, 3.4)	<0.0001
Physical activity								
≥30 min	Reference		Reference		Reference		Reference	
<30 min	1.9 (1.7, 2.2)	<0.0001	1.4 (1.1, 1.7)	0.007	2.1 (1.7, 2.6)	<0.0001	1.5 (1.1, 1.9)	0.008
Parity								
0	Reference		Not retained		Reference		Not retained	
≥1	1.0 (0.9, 1.2)	0.94			1.2 (0.9, 1.5)	0.13		
Preconception care								
No	Reference		Reference		Reference		Reference	
Yes	0.9 (0.8, 1.0)	0.028	1.0 (0.8, 1.2)	0.78	0.7 (0.6, 0.9)	0.001	0.9 (0.7, 1.2)	0.46
Previous Pregnancy complications								
No	Reference		Reference		Reference		Reference	
Yes	1.4 (1.2, 1.7)	<0.0001	1.2 (0.9, 1.5)	0.19	1.5 (1.2, 1.9)	0.001	1.3 (0.9, 1.7)	0.11
Use of OCP								
No	Reference		Reference		Reference		Not retained	
Yes	0.9 (0.7, 1.0)	0.018	0.8 (0.7, 1.0)	0.09	1.1 (0.9, 1.3)	0.32		
Family history of diabetes								
No	Reference		Reference		Reference		Reference	
Yes	2.1 (1.9, 2.4)	<0.0001	1.7 (1.4, 2.1)	<0.0001	2.6 (2.2, 3.1)	<0.0001	1.9 (1.4, 2.4)	<0.0001
BMI (kg/m^2^)		<0.0001		0.003		<0.0001		<0.0001
Underweight (<18.5)	0.6 (0.4, 0.9)		0.9 (0.5, 1.9)		0.6 (0.2, 1.5)		0.9 (0.3, 3.1)	
Normal weight (18.5–22.9)	Reference		Reference		Reference		Reference	
Overweight (23–24.9)	1.3 (1.0, 1.5)		1.5 (1.1, 2.0)		2.8 (2.0, 3.9)		1.8 (1.1, 2.9)	
Obese (≥25)	2.2 (1.9, 2.6)		1.7 (1.3, 2.2)		5.1 (3.8, 6.8)		2.9 (1.9, 4.3)	
HTN								
No	Reference		Not retained		Reference		Reference	
Yes	1.0 (0.9, 1.3)	0.68			1.5 (1.2, 1.9)	0.001	1.3 (0.9, 1.9)	0.17
Fruits intake								
7 days/week	Reference		Reference		Reference		Reference	
<7 days/week	0.6 (0.5, 0.7)	<0.0001	0.8 (0.6, 1.0)	0.08	0.6 (0.5, 0.7)	<0.0001	1.3 (0.9, 1.9)	0.21
Vegetable intake								
7 days/week	Reference		Reference		Reference		Reference	
<7 days/week	0.8 (0.7, 0.9)	0.002	1.0 (0.8, 1.2)	0.96	0.8 (0.7, 1.0)	0.048	0.8 (0.6, 1.1)	0.21

OR, odds ratio; CI, confidence interval; BDT, Bangladeshi Taka; BMI, body mass index; HTN, hypertension; OCP, oral contraceptive pill.

Adjusted odds ratios were estimated from multivariable logistic regression models including all retained covariates. Variables indicated as “Not retained” were excluded from the final multivariable model because they were not independently associated with the outcome. For variables with more than two categories (education, monthly household expenditure, and BMI), p-values represent overall Wald tests for the variable, whereas odds ratios are shown for individual categories relative to the reference category.

**Fig. 1:**
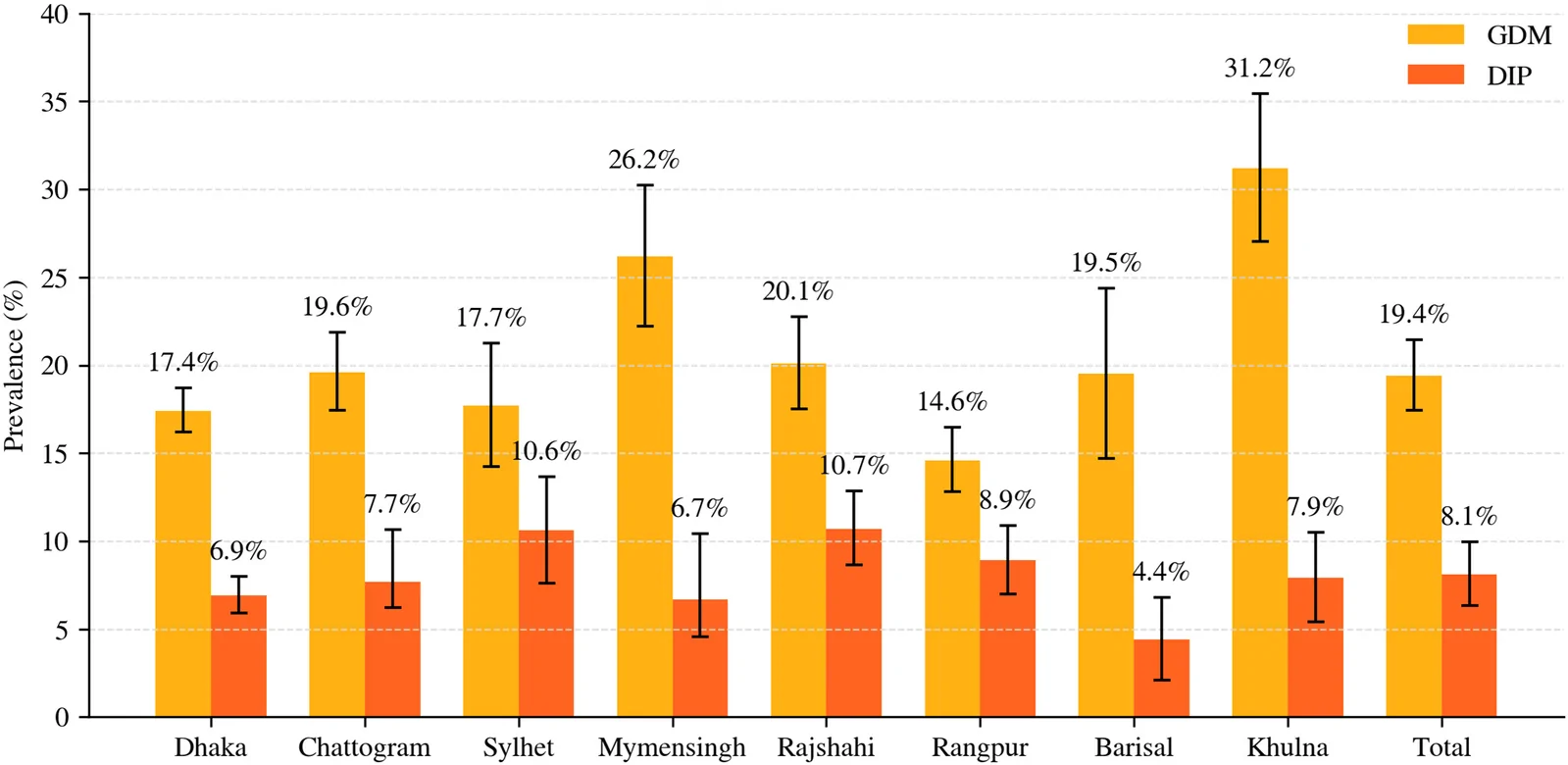
**Prevalence of gestational diabetes mellitus (GDM) and diabetes in pregnancy (DIP) by administrative division in Bangladesh.** Bars show the prevalence (%) of GDM and DIP among pregnant women attending antenatal care across the eight administrative divisions and overall. Error bars indicate 95% confidence intervals. Abbreviation: GDM, gestational diabetes mellitus; DIP, diabetes in pregnancy.

Overall prevalence was 19.4% (95% CI 18.4%–20.3%) for GDM and 8.1% (95% CI 7.4%–8.7%) for DIP. GDM prevalence was highest in Khulna (31.2%), followed by Mymensingh (26.2%) and Rajshahi (20.1%), and lowest in Rangpur (14.6%). DIP prevalence was highest in Rajshahi (10.7%), followed by Sylhet (10.6%) and Rangpur (8.9%) (Fig. 1).

**Fig. 2:**
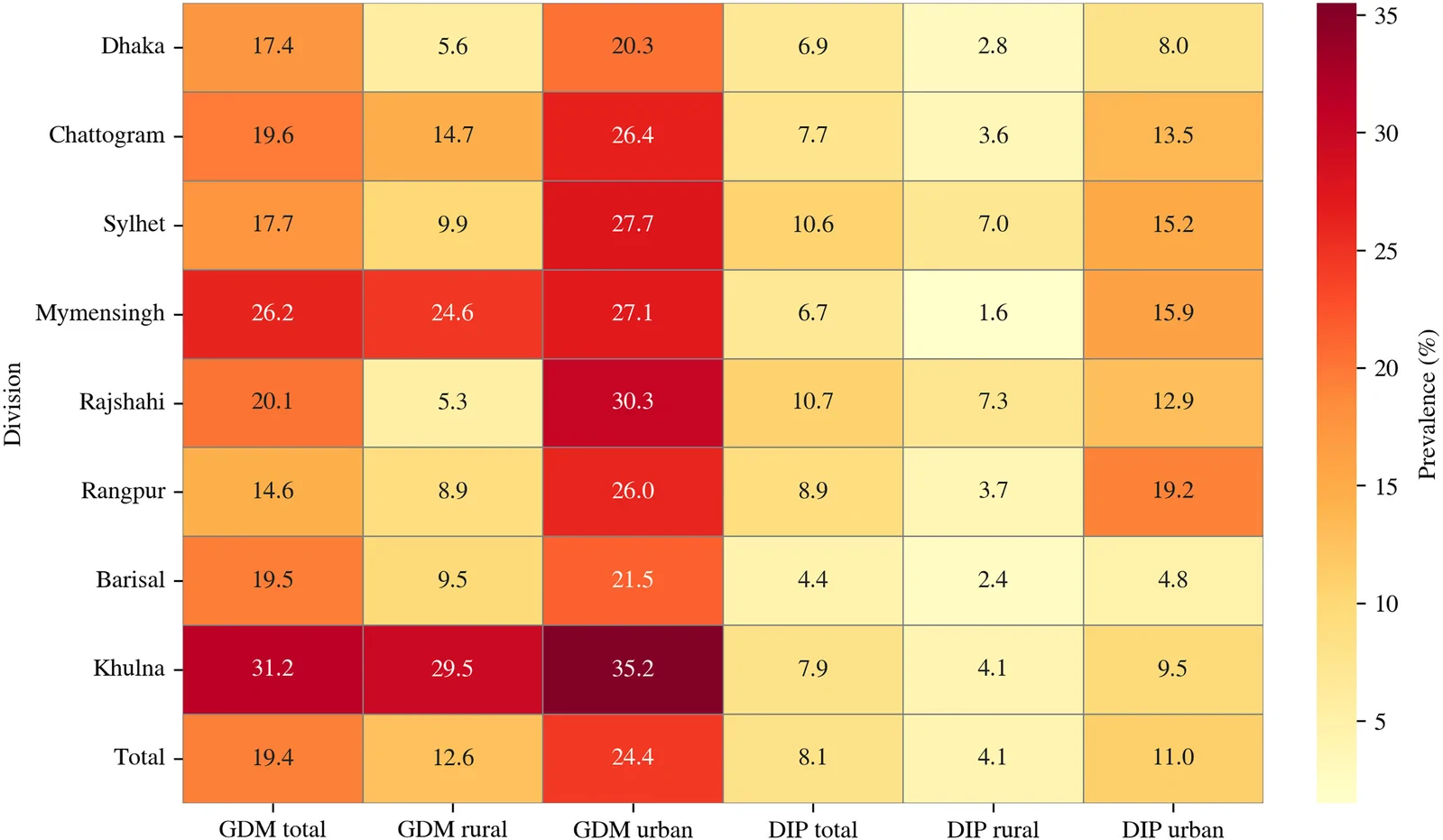
**Urban–rural distribution of gestational diabetes mellitus (GDM) and diabetes in pregnancy (DIP) across administrative divisions in Bangladesh.** Heatmap showing the prevalence (%) of GDM and DIP among pregnant women attending antenatal care across the eight administrative divisions, stratified by urban and rural residence, with overall totals. Colour intensity reflects prevalence, with darker shades indicating higher values. Abbreviation: GDM, gestational diabetes mellitus; DIP, diabetes in pregnancy.

There was consistently higher prevalence in urban than rural areas across all divisions. Nationally, GDM prevalence was 24.4% in urban areas compared with 12.6% in rural areas, while DIP prevalence was 11.0% and 4.1%, respectively. In Khulna, GDM prevalence was 35.2% in urban areas compared with 29.5% in rural areas, while in Rangpur, DIP prevalence was 19.2% in urban areas versus 3.7% in rural areas (Fig. 2).

**Fig. 3:**
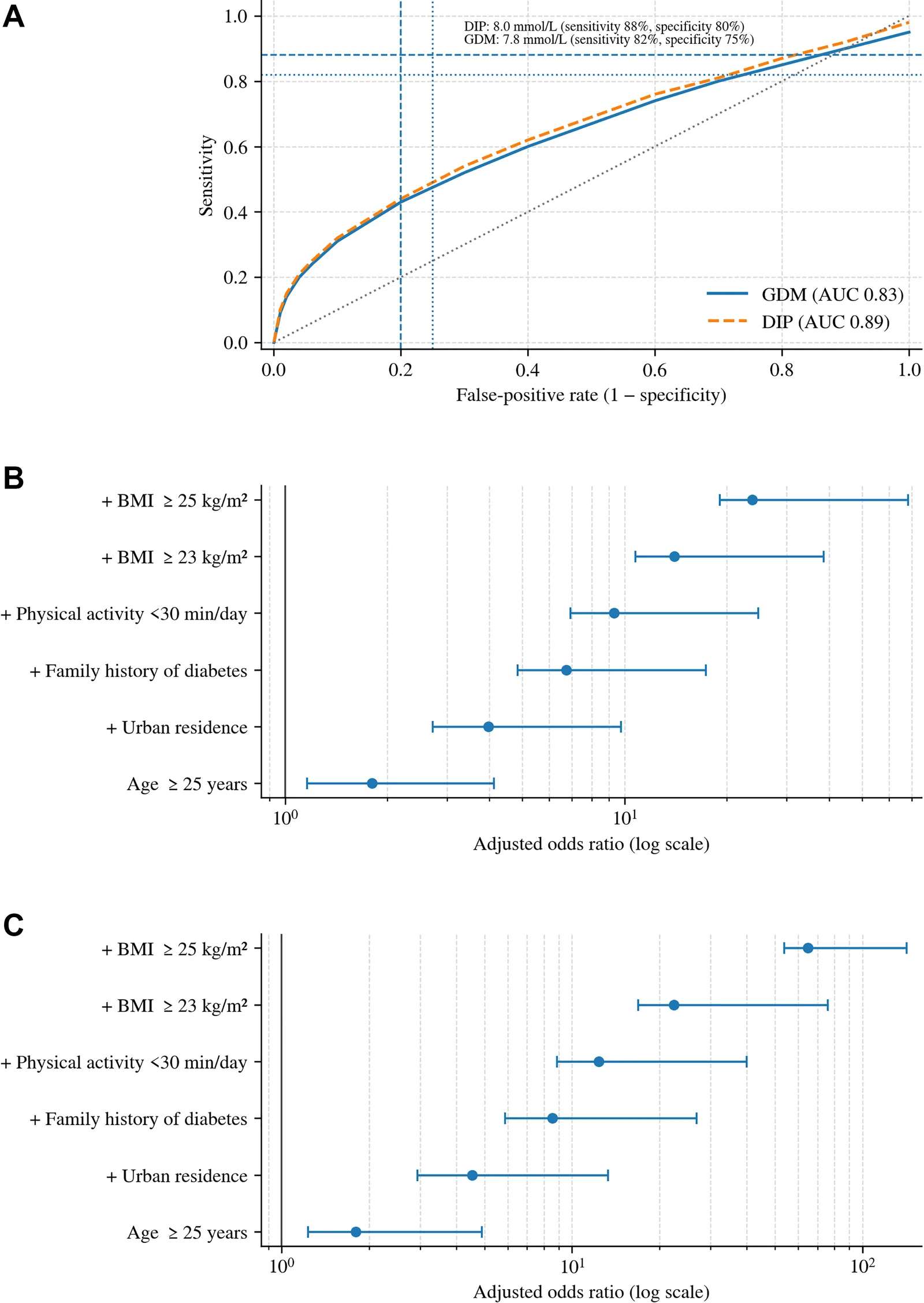
**Diagnostic performance of the glucose challenge test (GCT) and cumulative maternal factors associated with gestational diabetes mellitus (GDM) and diabetes in pregnancy (DIP).** (A) Receiver operating characteristic (ROC) curves showing the performance of the GCT for identifying GDM and DIP. The area under the curve (AUC) was 0.83 for GDM and 0.89 for DIP. Vertical and horizontal reference lines indicate the optimal cut-off values based on the Youden index, corresponding to 7.8 mmol/L for GDM (sensitivity 82%, specificity 75%) and 8.0 mmol/L for DIP (sensitivity 88%, specificity 80%). (B) Adjusted odds ratios for GDM associated with sequential addition of maternal factors, including age ≥25 years, urban residence, family history of diabetes, physical activity <30 min/day, BMI ≥23 kg/m^2^, and BMI ≥25 kg/m2. (C) Adjusted odds ratios for DIP associated with the same set of maternal factors. Odds ratios are presented on a logarithmic scale, and horizontal bars indicate 95% confidence intervals. Physical activity ≥30 min/day was used as the reference category. Abbreviation: GCT, glucose challenge test; GDM, gestational diabetes mellitus; DIP, diabetes in pregnancy; AUC, area under the curve.

The GCT demonstrated good discriminatory performance for both GDM and DIP, with AUCs of 0.83 and 0.89, respectively. The optimal GCT cut-off values were 7.8 mmol/L for GDM (sensitivity 82%, specificity 75%) and 8.0 mmol/L for DIP (sensitivity 88%, specificity 80%). At the optimal GCT cut-off of 7.8 mmol/L for GDM, the positive predictive value (PPV) was 45.2% and the negative predictive value (NPV) was 94.1%. For DIP at the optimal cut-off of 8.0 mmol/L, PPV was 28.4% and NPV was 98.3%. At the optimal cut-offs, NPV exceeded 94% for both outcomes, whereas PPV ranged from 28.4% to 45.2% (data not shown) (Fig. 3A).

Cumulative risk analyses for GDM and DIP is represented in Fig. 3B and C, respectively. A greater number of maternal risk factors was associated with progressively higher odds of both GDM and DIP. Odds increased with the sequential accumulation of older age, urban residence, family history of diabetes, low physical activity (<30 min/day), and increasing BMI. For GDM, cumulative odds increased notably with overweight and obesity, whereas for DIP, obesity was associated with a steeper escalation of cumulative risk.

## Discussion

This study is a cross-sectional analysis of 6631 pregnant women screened at 24–28 weeks’ gestation at BADAS centres, to estimate the prevalence of GDM % and DIP yielding a combined HIP burden of 27.5%. Older maternal age (≥25 years), higher BMI, family history of diabetes, low physical activity, and urban residence were independently associated with both outcomes. Prevalence varied across administrative divisions, and within every division, urban prevalence exceeded rural prevalence. The 50-g GCT demonstrated good discriminatory performance for both outcomes, with empirically derived optimal thresholds close to 7.8 mmol/L for GDM and 8.0 mmol/L for DIP. These findings are consistent with observed patterns in cardiometabolic risk factors and should be interpreted as associations rather than causal relationships.

Placed against the Bangladesh literature, the national estimate for GDM (19.4%) is higher than the pooled estimate of 13% (95% CI 7–21) reported in a Bangladesh-specific systematic review and meta-analysis synthesising eight studies.^4^ That review identified older age and higher BMI as key correlates, patterns reproduced in the present study using a larger and geographically comprehensive sample. Earlier studies in Bangladesh were frequently single-centre, urban, and hospital-based and applied heterogeneous diagnostic criteria, which likely contributed to wide variation in reported prevalence and limited generalisability. By contrast, the standardised procedures and national coverage of the present analysis provide internally consistent estimates. However, as this was a facility-based study, generalisability to the entire pregnant population of Bangladesh should be made with caution, particularly for women who do not attend antenatal care. Our findings provide clearer quantification of previously suggested urban–rural differences.^4^^,^^10^, ^11^, ^12^

The heterogeneity in reported GDM prevalence in Bangladesh likely reflects differences in sampling frames, screening strategies, and diagnostic thresholds. Adoption of the IADPSG/WHO-2013 criteria has consistently been associated with higher case detection compared with earlier definitions.^4^, ^5^, ^6^ In the present study, a pragmatic two-step pathway—GCT triage followed by OGTT interpreted using IADPSG/WHO-2013 criteria—was applied to balance feasibility with diagnostic sensitivity, consistent with practice in many low- and middle-income settings. However, this approach may underestimate true prevalence, as women with a negative GCT were not referred for confirmatory OGTT.

The optimal screening strategy for GDM remains debated. Although IADPSG/WHO recommends a one-step 75-g OGTT, a large, randomised community-based trial (n = 28,771) comparing one-step versus two-step screening reported higher detection with the one-step approach (9.3% vs 5.4%; p < 0.001) without corresponding differences in key maternal or neonatal outcomes.^24^ These findings suggest that increased diagnostic yield may not necessarily translate into improved short-term clinical outcomes. In resource-constrained health systems such as Bangladesh, where laboratory capacity is limited and patient volumes are high, a two-step strategy may offer a pragmatic balance between diagnostic yield and feasibility. The initial GCT triage substantially reduces the number of OGTTs required, facilitating implementation of screening at scale.

Comparisons with neighbouring South Asian populations further contextualise these findings. In India, OGTT-based studies report associations between GDM and maternal age, adiposity, and family history, while urban–rural differences are variably attenuated after adjustment.^5^ Beyond India, studies from Pakistan and Sri Lanka demonstrate similar patterns linking adiposity and physical inactivity with GDM.^25^^,^^26^ Despite differences in design and context, the direction and magnitude of associations are broadly consistent, supporting the plausibility of the observed findings in Bangladesh. Systematic reviews from Eastern and South-Eastern Asia similarly report substantial heterogeneity driven by diagnostic criteria and urbanisation.^6^

From a global perspective, the IDF Diabetes Atlas (11th edition) estimates that approximately one in five pregnancies worldwide are affected by HIP, with South-East Asia among the highest-burden regions.^3^ The estimates reported here fall within this range, underscoring the scale of screening and follow-up required. Evidence from the Hyperglycemia and Adverse Pregnancy Outcome (HAPO) study linking graded maternal hyperglycaemia to adverse outcomes further reinforces the importance of detection in high-burden settings.^1^

Urban–rural and division-level differences warrant careful interpretation. Higher urban prevalence likely reflects both compositional factors (older age, higher BMI, socioeconomic position) and contextual influences, including dietary transition and reduced physical activity. Prior evidence based predominantly on urban hospital populations may have underestimated these gradients.^4^^,^^10^, ^11^, ^12^ The persistence of urban–rural differences after adjustment suggests potential contextual influences that merit further investigation.

Beyond the urban–rural gradient, variation across urban centres was also observed. For example, urban GDM prevalence was higher in Khulna than in Dhaka. These differences may reflect heterogeneity in population characteristics, lifestyle patterns, and healthcare utilisation across cities, including differences in diet, physical activity, and screening uptake. However, given the facility-based design and smaller division-level sample sizes, some variation may also be attributable to sampling variability. These findings highlight the importance of subnational analyses to better understand geographic variation and inform context-specific prevention and service delivery strategies.

The screening implications of these findings are clinically relevant. The two-step screening pathway demonstrated good discrimination, supporting its use where universal OGTT screening is not feasible. A GCT threshold around 7.8 mmol/L may represent a pragmatic balance between sensitivity and specificity in resource-constrained settings.

The clinical and life-course implications are substantial. GDM is associated with adverse pregnancy outcomes and increased long-term cardiometabolic risk for both mother and offspring.^1^^,^^2^ By quantifying national burden and identifying high-prevalence subgroups, the present study provides evidence to inform antenatal counselling, weight management, and postpartum follow-up strategies.

This study has several strengths, including nationwide coverage across all administrative divisions of Bangladesh, a large sample size, and standardised data collection procedures. Prespecified multivariable analyses, laboratory quality control, and uniform application of IADPSG/WHO-2013 criteria strengthen internal validity and comparability.

Several limitations should be acknowledged. The cross-sectional design precludes causal inference and does not allow assessment of postpartum glycaemic trajectories or long-term maternal and offspring outcomes. Although associations between maternal factors and GDM or DIP were identified, these should not be interpreted as causal and may be subject to residual confounding. Furthermore, data on ethnicity and gender identity were not collected; therefore, potential differences in hyperglycaemia in pregnancy across ethnic or gender groups could not be evaluated.

The two-step screening strategy (GCT followed by OGTT only among screen-positive women) may have introduced partial verification bias, potentially underestimated true prevalence while influencing estimates of diagnostic performance. Evidence from a large, randomised trial suggests that one-step screening identifies more cases without clear differences in short-term outcomes.^24^ In this context, the two-step approach used in the present study reflects a pragmatic compromise between diagnostic completeness and feasibility in resource-constrained settings.

Data on intolerance to glucose loading (e.g., vomiting during GCT or OGTT) were not systematically collected; therefore, exclusions due to intolerance and comparative tolerability between the 50-g and 75-g protocols could not be assessed.

Participants were recruited from BADAS-affiliated antenatal centres using a facility-based design rather than population-based sampling. Although BADAS provides services to a broad population across urban and rural settings, and the study included substantial rural representation, the absence of sampling weights and lack of adjustment for clustering at the centre or district level may affect the precision of estimates and limit full national representativeness.

Measurement variability is possible. Initial screening relied on capillary glucometer measurements, whereas diagnosis was based on venous plasma glucose processed across multiple laboratories. Although prior validation studies in Bangladesh demonstrate strong concordance between capillary and venous measurements, systematic differences cannot be entirely excluded.^18^ In addition, behavioural variables were self-reported, and BMI was measured at mid-gestation rather than pre-pregnancy, which may have resulted in non-differential misclassification and attenuation of associations.

Screening was conducted at 24–28 weeks’ gestation in accordance with current guidelines; therefore, early gestational diabetes diagnosed before 20 weeks was not assessed.

Finally, heterogeneity in diagnostic criteria and study designs across Bangladesh and the wider region limits direct comparability with earlier estimates, although the use of a standardised protocol in the present study improves internal consistency.^3^^,^^4^^,^^6^^,^^10^, ^11^, ^12^

In conclusion, hyperglycaemia in pregnancy affects a substantial proportion of women attending antenatal care in Bangladesh, with clear urban–rural and geographic variation. Older age, higher BMI, family history of diabetes, physical inactivity, and urban residence were consistently associated with both GDM and DIP.

These findings support the integration of standardised screening of hyperglycemia in pregnancy into routine antenatal care in high-burden settings, alongside strategies addressing modifiable maternal factors.

## Contributors

B.B. conceptualised the study, designed the methodology, supervised the project, conducted the formal analysis, wrote the original draft, and is the guarantor. T.S. curated and formally analysed the data, prepared visualisations, and wrote the original draft. S.P., L.Q.N., S.A., S.K., F.A., T.A., F.P., S.J.S., K.H., A.K., S.S., and K.A.C. conducted the investigation, administered the project, and curated data. M.H.J. and A.H.M.E.H. reviewed and edited the manuscript. H.M. and A.K.A.K. conceptualised the study, provided resources, and supervised the project. B.B. and T.S. directly accessed and verified the underlying data. B.B. had final responsibility for the decision to submit the manuscript. All authors reviewed and edited the manuscript, approved the final version, and accept responsibility for the work.

## Data sharing statement

De-identified individual participant data, the data dictionary, study protocol, and analysis code are available from the corresponding author (B.B.) on reasonable request, subject to approval by the Diabetic Association of Bangladesh (BADAS) Ethical Review Committee and a data access agreement. Materials will be available beginning at publication and for at least 5 years thereafter.

## Editor note

The Lancet Group takes a neutral position with respect to territorial claims in published maps and institutional affiliations.

## Declaration of interests

The authors declare no competing interests.

## References

[1] Metzger B.E., Lowe L.P., Dyer A.R., HAPO Study Cooperative Research Group (2008). Hyperglycemia and adverse pregnancy outcomes. N Engl J Med.

[2] Bellamy L., Casas J.P., Hingorani A.D., Williams D. (2009). Type 2 diabetes mellitus after gestational diabetes: a systematic review and meta-analysis. Lancet.

[3] International Diabetes Federation (2024).

[4] Begum R., Roy S., Banik S. (2022). The prevalence of gestational diabetes mellitus in Bangladesh: a systematic review and meta-analysis. Int J Diabetes Dev Ctries.

[5] Bhavadharini B., Mahalakshmi M.M., Anjana R.M. (2016). Prevalence of GDM in urban and rural Tamil Nadu using IADPSG and WHO-1999 criteria (WINGS-6). Clin Diabetes Endocrinol.

[6] Nguyen C.L., Pham N.M., Binns C.W. (2018). Prevalence of gestational diabetes mellitus in Eastern and South-Eastern Asia: a systematic review and meta-analysis. J Diabetes Res.

[7] World Bank (2026). Urban Population (% of Total Population) – Bangladesh. World Bank Development Indicators. https://databank.worldbank.org/source/world-development-indicators.

[8] Afsana F., Bhowmik B., Siddiquee T. (2021). Current practices in diagnosis and management of gestational diabetes: a Bangladesh study. J Diabetol.

[9] Bear A.P., Bennett W.L., Katz J. (2023). Self-reported diabetes or hypertension diagnoses and antenatal care among child-bearing women in rural Bangladesh: a cross-sectional study. PLOS Glob Public Health.

[10] Jesmin S., Akter S., Akashi H. (2014). Screening for gestational diabetes mellitus and its prevalence in Bangladesh. Diabetes Res Clin Pract.

[11] Sayeed M.A., Mahtab H., Khanam P.A. (2005). Diabetes and hypertension in pregnancy in a rural community of Bangladesh: a population-based study. Diabet Med.

[12] Mazumder T., Akter E., Rahman S.M. (2022). Prevalence and risk factors of gestational diabetes mellitus in Bangladesh: findings from demographic health survey 2017-2018. Int J Environ Res Public Health.

[13] World Health Organization (2025). STEPwise approach to NCD risk factor surveillance (STEPS) [WWW Document]. https://www.who.int/teams/noncommunicable-diseases/surveillance/systems-tools/steps.

[14] Bhowmik B., Siddiquee T., Ahmed T. (2021). Diabetes care during 50 years of Bangladesh. J Diabetol.

[15] Vandenbroucke J.P., von Elm E., Altman D.G. (2007). Strengthening the reporting of observational studies in epidemiology (STROBE): explanation and elaboration. PLoS Med.

[16] International Association of Diabetes and Pregnancy Study Groups (IADPSG) (2010). Recommendations on the diagnosis and classification of hyperglycaemia in pregnancy (Consensus Panel). Diabetes Care.

[17] (2013). Diagnostic Criteria and Classification of Hyperglycaemia First Detected in Pregnancy.

[18] Bhowmik B., Siddiquee T., Munir S.B. (2025). Random capillary blood glucose in the diagnosis of diabetes: a cross-sectional study in Bangladesh. BMJ Open.

[19] Choo V. (2002). WHO reassesses appropriate body-mass index for Asian populations. Lancet.

[20] Whelton P.K., Carey R.M., Aronow W.S. (2018). 2017 ACC/AHA/AAPA/ABC/ACPM/AGS/APhA/ASH/ASPC/NMA/PCNA guideline for the prevention, detection, evaluation, and management of high blood pressure in adults: a report of the American college of cardiology/American heart association task force on clinical practice guidelines. J Am Coll Cardiol.

[21] Bangladesh Bureau of Statistics Population and housing census- 2022. http://www.bbs.org.bd.

[22] Khan M.M.H., Aklimunnessa K., Kabir M.A. (2006). Determinants of physical activity among urban adults in Bangladesh: a case- control study. J Phys Act Health.

[23] Moniruzzaman M., Ahmed M.S., Zaman M.M. (2019). Physical activity levels and associated factors among adults in rural Bangladesh: a cross- sectional study. BMC Public Health.

[24] Ramezani Tehrani F., Rahmati M., Farzadfar F. (2023). One-step versus two-step screening for diagnosis of gestational diabetes mellitus in Iranian population: a randomized community trial. Front Endocrinol.

[25] Iqbal R., Rafique G., Badruddin S. (2007). Increased body fat percentage and physical inactivity are independent predictors of gestational diabetes mellitus in South Asian women. Eur J Clin Nutr.

[26] Kande Vidanalage C.J., Senarath U., Silva K.D. (2016). Effects of initial BMI on development of gestational diabetes in a rural Sri Lankan population: a case-control study. Diabetes Metab Syndr.

