# Supplementary material for: Prevalence of and factors associated with gestational diabetes mellitus and diabetes in pregnancy in Bangladesh: a facility-based cross-sectional study (2019–2020)
Source: Lancet Reg Health Southeast Asia. 2026 Sep 10;54:100861. doi: 10.1016/j.lansea.2026.100861 (PMC13584127; doi:10.1016/j.lansea.2026.100861)
Supplement: Supplementary file 1 [file mmc1.docx]

| **Supplement document** | **Page** |
| --- | --- |
| Supplementary Table 1. Prevalence of GDM and DM in Pregnancy (DIP) among the study participants | 2 |

| **Supplementary Table 1.** **Prevalence of GDM and DM in Pregnancy (DIP) among the study participants** | | | | |
| --- | --- | --- | --- | --- |
| **Variable** | **GDM (n= 1285)** | **P value** | **DIP (n= 534)** | **P value** |
| Age group |  | <0.001 |  | <0.001 |
| - <25 years | 12.8 (383) |  | 4.2 (126) |  |
| - ≥25 years | 24.8 (902) |  | 11.2 (408) |  |
| Education (years) |  | <0.001 |  | <0.001 |
| - 0-5 | 15.3 (253) |  | 5.8 (96) |  |
| - 6-10 | 18.8 (514) |  | 7.6 (208) |  |
| - >10 | 23.7 (457) |  | 9.8 (189) |  |
| Occupation |  | 0.003 |  | 0.016 |
| - Housemaker | 22.3 (290) |  | 7.8 (416) |  |
| - Others | 18.7 (995) |  | 9.1 (118) |  |
| Monthly expense (BDT) |  | <0.001 |  | <0.001 |
| - Low income (<10000) | 15.4 (400) |  | 5.6 (144) |  |
| - Middle income (10000-20000) | 19.7 (558) |  | 8.9 (252) |  |
| - High income (>2000) | 28.8 (272) |  | 11.8 (111) |  |
| Location |  | <0.001 |  | <0.001 |
| - Rural | 12.6 (353) |  | 4.1 (115) |  |
| - Urban | 24.4 (932) |  | 11.0 (419) |  |
| Physical activity |  | <0.001 |  | <0.001 |
| - ≥30 minutes | 15.1 (458) |  | 6.0 (182) |  |
| - < 30 minutes | 23.7 (663) |  | 10.4 (292) |  |
| Number of parities |  | 0.943 |  | 0.136 |
| - 0 | 20.8 (543) |  | 8.7 (226) |  |
| - ≥1 | 20.9 (327) |  | 10.0 (157) |  |
| Preconception care |  | 0.080 |  | 0.004 |
| - No | 20.6 (435) |  | 9.6 (202) |  |
| - Yes | 18.7 (768) |  | 7.4 (305) |  |
| Previous Pg complications |  | 0.001 |  | 0.009 |
| - No | 19.8 (600) |  | 8.6 (260) |  |
| - Yes | 24.8 (245) |  | 11.3 (112) |  |
| Use of OCP |  | 0.011 |  | 0.173 |
| - No | 20.2 (746) |  | 7.7 (284) |  |
| - Yes | 17.6 (418) |  | 8.7 (206) |  |
| Family history of diabetes |  | <0.001 |  | <0.001 |
| - No | 15.8 (684) |  | 6.0 (261) |  |
| - Yes | 26.4 (573) |  | 12.3 (267) |  |
| BMI (kg/m^2^) |  | <0.001 |  | <0.001 |
| - Underweight (<18.5) | 9.5 (24) |  | 2.0 (5) |  |
| - Normal weight (18.5-22.9) | 14.6 (258) |  | 3.0 (53) |  |
| - Overweight (23-24.9) | 17.0 (217) |  | 7.6 (97) |  |
| - Obese (≥25) | 24.7 (722) |  | 11.7 (342) |  |
| HTN |  | 0.944 |  | 0.001 |
| - No | 19.4 (1122) |  | 7.6 (442) |  |
| - Yes | 19.3 (163) |  | 10.9 (92) |  |
| Fruits intake |  | <0.001 |  | 0.384 |
| - 7 days/ week | 18.1 (1035) |  | 8.8 (79) |  |
| - <7 days/ week | 27.8 (250) |  | 7.9 (456) |  |
| Vegetable intake |  | 0.002 |  | <0.001 |
| - 7 days/week | 18.4 (868) |  | 7.1 (333) |  |
| - <7 days/week | 21.8 (417) |  | 10.5 (201) |  |
| Data is presented as percentage (number). Abbreviation: BDT, Bangladeshi taka (1 USD= 120 BDT); BMI, body mass index; DM, diabetes mellitus; GDM, gestational diabetes mellitus; HTN, hypertension; OCP, oral contraceptive pill. | | | | |

**Supplementary Figure 1. Urban–rural distribution of gestational diabetes mellitus (GDM) and diabetes in pregnancy (DIP) across administrative divisions**

Heatmap illustrating the prevalence (%) of gestational diabetes mellitus (GDM) and diabetes in pregnancy (DIP) across the eight administrative divisions of Bangladesh, stratified by rural and urban residence. Color intensity represents increasing prevalence, with darker shades indicating higher proportions. Division-wise totals and national averages are shown for comparison.
